# Using digital technologies to diagnose in the home: recommendations from a Delphi panel

**DOI:** 10.1038/s41746-024-01009-8

**Published:** 2024-01-22

**Authors:** David A. Simon, Sara Raza, Carmel Shachar, I. Glenn Cohen

**Affiliations:** 1 Northeastern University School of Law, Boston, USA; 2 Health Law and Policy Clinic, Harvard Law School, Cambridge, USA; 3 Petrie-Flom Center for Health Law Policy, Biotechnology & Bioethics, Harvard Law School, Cambridge, USA

**Keywords:** Law, Decision making

## Abstract

Rapid advances in digital technology have expanded the availability of diagnostic tools beyond traditional medical settings. Previously confined to clinical environments, these many diagnostic capabilities are now accessible outside the clinic. This study utilized the Delphi method, a consensus-building approach, to develop recommendations for the development and deployment of these innovative technologies. The study findings present the 29 consensus-based recommendations generated through the Delphi process, providing valuable insights and guidance for stakeholders involved in the implementation and utilization of these novel diagnostic solutions. These recommendations serve as a roadmap for navigating the complexities of integrating digital diagnostics into healthcare practice outside traditional settings like hospitals and clinics.

## Introduction

Digital technologies are advancing at a rapid pace, pushing diagnostics into smaller and more portable devices—many of them outside the traditional healthcare settings. For example, patients can record their sleep patterns using an Apple Watch or FitBit^1,2^, “touchlessly” monitor their stress and blood pressure using the phone application veyetals^3^, or monitor their blood glucose levels in real time with a small device (such as the Freestyle Libre 2) that displays results on a companion phone application^4^. These are just a few examples of the many developers introducing products that use smartphones to record health-related information, such as mood, balance, sleep, and respiratory patterns. While these innovations are patient-centric, we are also seeing many such products designed to be used by physicians, such as a small portable ultrasound device that plugs into a smartphone and provides high-quality images instantly without the need for cumbersome equipment^5^.

We call this product category, which ranges from unregulated general wellness products to regulated devices, *in-home digital diagnostics* (or *digital diagnostics* for short). Box 1 provides a formal description of how we define the category for the purpose of this study.

Thus far, this product category has been subject to a confusing and incomplete patchwork of regulation by federal law and agencies, as well as other areas of state law, such as tort and contract law^6^. Some of these issues have already drawn the attention of interested parties, such as clinicians, academics, and regulators^7^. The Food and Drug Administration (FDA), for example, has attempted to respond to issues raised by digital diagnostics through guidance documents and formal programs, including its recent guidance on the difference between regulated devices and unregulated general wellness products^8^. It has also implemented a pilot program, which it completed in September 2022, to bring software as a medical device to market faster by evaluating firms rather than products^9^.

Legal uncertainty is also coupled with ethical uncertainty about the obligations of physicians, manufacturers, and society have toward those that use digital diagnostics. In other words, it is not clear how actors—patients, physicians, manufacturers, marketers—ought to behave, legally or ethically, when developing, implementing, prescribing, using, and paying for digital diagnostics. For example, what is the best way for manufacturers to communicate to patients how the digital diagnostic should be used? How should manufacturers protect patient privacy? What should physicians know about how the digital diagnostic uses or shares patient information? Do physicians have an ethical obligation to use or not use new technologies? How is this obligation complicated by provider and patients’ ability to access and pay for the digital diagnostic? Will insurance companies cover digital diagnostics and, if so, how much will they reimburse for them? If a digital diagnostic’s features change, how should patient consent be obtained? Among the issues these questions raise are those related to patient data privacy and consent, as well as the ethical obligations of those collecting, using, or recommending the use of digital diagnostics.

To answer these kinds of legal and ethics questions, we conducted a Delphi study (Box 2) to develop recommendations around digital diagnostics for developers, regulators, and public and private insurers. The Delphi technique was chosen because it is recognized as an optimal method for consensus building, with use of anonymous feedback from an expert panel and statistical analysis techniques to interpret the data. The iterative nature of the process avoids some of the pitfalls of other methods, such as the effects of dominant persons or the tendency to conform to a particular viewpoint^10^.

The Delphi brought together 19 experts with diverse experience—including founders of digital health technology companies, academics, practicing lawyers at leading technology and insurance companies, physicians, and entrepreneurs—and asked them how to balance these risks against the promise of digital diagnostics. Our study began with over 100 policy recommendations, which the authors drafted based on a review of the literature and prior work in this field. After three rounds of participant evaluation, the total number of consensus recommendations was 29. These recommendations fell into five domains: (1) guidelines, certification, and training relating to the use of digital diagnostics; (2) liability arising from the use of digital diagnostics; (3) the regulation and marketing of digital diagnostics; (4) reimbursement of digital diagnostics; and (5) privacy, security, and consent in the use of digital diagnostics. We intend that these recommendations serve as forward-looking issue-based guideposts for legislators, regulators, developers, payers, and users of digital diagnostics.

Box 1 Definition of in-home digital diagnostics ***In-Home:*** **outside of traditional healthcare settings**.

- Our definition excludes traditional healthcare settings include physician offices, brick-and-mortar hospitals, medical centers, and stand-alone testing facilities. On the other hand, an at-home sleep apnea testing device such as WatchPAT® would qualify as “in-home,” as would a smartphone application like Hyfe, which produces a cough report by tracking user cough patterns whenever the user initiates the app. As we use the term, “in-home” might also include a traditional healthcare service, such as an office visit, if performed remotely through video or telephone.

***Digital:*** **significantly incorporates a novel, technology-enabled component not traditionally found in diagnostic devices**.

- A self-testing kit – such as a pregnancy, ovulation, or drug abuse detection test that allows users to view results online – would not satisfy this definition of “digital” since the digital component does not significantly alter the analog self-test. By contrast, SpectraPass, which uses machine learning algorithms and a mass spectrometer’s laser on a protein sample to determine whether a patient is positive or negative for COVID-19, enabling adaptation of existing technology already deployed in hospital systems, would fall within our definition of “digital.” This flexible definition captures the breadth of technologies where the digital component *significantly changes* the nature of the device.

***Diagnostics:*** **any device that can aid in the identification of a particular disease or condition, or event associated with that disease or condition**.

- This definition covers the initial diagnosis and subsequent events caused by a particular disease or condition. Glucose monitors, for example, would fit within this definition because they can aid in the diagnosis of low blood sugar even though a patient typically uses one only *after* an initial diabetes diagnosis.

Our project focuses on products that meet all three criteria.

Box 2 What is a Delphi? Pioneered by the RAND corporation to forecast the effect of technology on warfare, the Delphi is a method for developing consensus among experts^16^. Since its inception, it been used to study issues ranging from drug and device regulation^27^, to nursing^28^, to clinical decision making^29^ and bioethics^30^. The technique can vary according to context and objectives. In general, however, the Delphi identifies and solicits participation from experts to help develop consensus around a particular issue through some combination of asynchronous and synchronous surveys that ask participants to evaluate recommendations. Criteria are developed to eliminate recommendations that do not reach consensus or to select those that reach strong consensus.

## Methods

### General summary of Delphi process

In rounds 1 and 2, the project team engaged key stakeholders through individual interviews, case studies, and focus groups and formulated the following 5 domains as in need of further guidance: (1) guidelines, certification, and training; (2) liability; (3) regulation and marketing; (4) reimbursement; and (5) privacy, security, and consent. These domains were identified through prior work with the Diagnosing in the Home project through conversations with stakeholders, and in consultation with the steering committee for the project^11^. Each category is meant to identify a distinct class of conduct. For example, “regulation” could plausibly be read to include “guidelines and certification.” In this study, however, we use the term “regulation” to mean formal administrative or legal action by a legislature or formal regulator. While certification and guidelines may be included in a regulatory scheme, in this study those terms are taken primarily to mean actions by private parties not otherwise sanctioned by the government or formal regulator.

We, the authors of this study, focused on developing recommendations for actors that are most important within each domain. For example, recommendations on the first domain, guidance, certification, and training, are directed to medical organizations, physicians, manufacturers, caregivers, and licensure, accreditation, and standard-setting bodies.

To identify recommendations that best addressed the ethical and regulatory implementation of digital diagnostics, we then employed a modified Delphi process, which uses multiple rounds of evaluation to gauge and facilitate consensus among a group of expert stakeholders on a particular topic^12–15^. Using a three-round process—two asynchronous online surveys and one synchronous video conference—the participants voted on candidate recommendations. Each round, consensus criteria were used to eliminate candidate recommendations and advance those remaining to the next round. Round 1 began with over 100 recommendations and asked participants to provide qualitative feedback on the clarity, importance, and correctness of the proposed recommendations and suggestions for additional recommendations. This yielded 54 recommendations, which participants rated in Round 2 along three axes: need, correctness, and feasibility. We choose these categories because our study aimed to develop policy recommendations capable of responding to a real challenge (need), providing appropriate guidance (correctness), and being successfully implemented (feasibility). While 20 of the 54 recommendations met the overall criteria for consensus and were deemed “accepted” without further discussion, 12 recommendations exhibited some level of disagreement. In Round 3 participants discussed the disagreements about these 12 recommendations during a synchronous video call, refining some and eliminating others.

A full list of recommendations and a full list of panelists are provided in Supplementary Information. Below we explain the methods of our Delphi in more detail, focusing on how each round of the Delphi was conducted, the decision rules for developing consensus, and the use of criteria to select recommendations in each round.

### The rounds of the Delphi

The 19 members of our Delphi expert panel were selected with the aim of reflecting the diversity of stakeholders involved in the development and use of digital diagnostics, without seeking representativeness given the small size of the group, as is typical. Members included patients, patient advocates, nurses, physicians, medical officers, venture capitalists, product developers, data scientists, experts in bioethics, and experts in law (see Supplementary Table 1) for a complete list of participants). Participants were selected based on their proven expertise in these areas, as exhibited by publication record and professional position, and reputation. Once an individual agreed to participate, suggestions for further well-qualified participants were solicited from them, which were used to inform subsequent choices about whom to invite.

Our Delphi process consisted of 3 rounds. Before the first round, the project team drafted over 100 policy-level recommendations responsive to the 5 domains and targeted at actors identified as relevant to each domain. In round 1, we began our survey with five open-ended questions that asked participants about (i) the most important ethical or legal issues facing the development and implementation of digital diagnostics; (ii) the ethical and legal challenges that make digital diagnostics different and unique from other diagnostic devices used in traditional clinical settings, (iii) the legal and ethical challenges that stakeholders experienced with designing, manufacturing, or marketing of digital diagnostics, (iv) the legal and ethical challenges that stakeholders experienced with incorporating digital diagnostics into their medical experience, and (v) the legal and ethical challenges that digital diagnostics present. We asked panel members to provide qualitative feedback on the clarity, importance, and correctness of the proposed recommendations and suggestions for additional recommendations. This resulted in 54 recommendations for evaluation in round 2.

In round 2, members of the Delphi panel were asked to complete a survey evaluating the 54 recommendations along 3 axes: need, correctness, and feasibility. The choice of axes was motivated by our aim of selecting policy recommendations capable of responding to a real challenge (need), providing appropriate guidance (correctness), and being successfully implemented (feasibility). The criteria for determining consensus were based on the UCLA/RAND consensus criteria adjusted for the size of our panel^16^. Twenty of the 54 recommendations met the overall criteria for consensus after round 2 voting and were thus deemed “accepted” without further discussion.

Round 3 consisted of a half-day video conference devoted to discussing approximately 12 recommendations that exhibited some level of disagreement on feasibility. Before the video conference, participants received information sheets summarizing the voting process, the results, and the recommendations that had been accepted, as well as those that had been discussed. During the video conference, the attending participants (which totaled 15) debated revisions to recommendations after moderated discussion among the group. Our process allowed for and encouraged changes to the wording and substance of the recommendations. Because our Round 2 produced such high levels of agreement on recommendations, and because not all panelists could participate in Round 3, we used the videoconference to discuss modifications to the identified recommendations.

Because revisions were quite minor, the project team sent participants the changes and asked to respond if they did not agree with the modified recommendations. A lack of response within one week signified acceptance.

### Decision rules for consensus

In any Delphi process, decision rules are determined in advance to both define and determine consensus. Consensus on a topic is usually determined if a certain number or percentage of the votes falls within a prescribed range. It is best to determine our criteria for consensus a priori to avoid bias.

We constructed a panel size of *n* = 19. The Panel used two different 7-vote scales for each round of the Delphi. The justification for the two different scales is the high number of initial recommendations (119). We explained to the panel both the scaling mechanism and the reason for the two different scales. Round 1 was primarily directed towards winnowing the pool of recommendations. For that reason, the panel used a 7-vote scale to measure three axes of opinions about each recommendation, where 7 represented positive views (importance, correctness, and clarity) and 1 represented negative views.

Round 2 was directed towards forming consensus on the recommendations remaining after Round 1. The panel used a 7-vote scale to measure three axes of opinions about each recommendation, where 7 represented positive views (correctness, high need, or feasibility) and 1 represented negative views (incorrect, low need, infeasible). Generally, endorsement of a recommendation was determined by high end (positive) scores without disagreement.

We based our criteria on the European Union BIOMED Concerted Action on Appropriateness for surgical procedures as referenced in The RAND/UCLA Appropriateness Method User’s Manual^17^. For clinical appropriateness determinations, one needs to determine agreement around appropriate, inappropriate, and equivocal designations, since any clinical scenario may occur and will need to be categorized. Furthermore, appropriateness studies assume there will be variation. Policy recommendations, while not absolute, are not intended to be empirically applied, so we sought greater agreement. Furthermore, in our case, we were concerned only with a decision about whether to accept or reject a recommendation. Therefore, we needed to focus on consensus around high scores.

We defined consensus (i.e., agreement), as a clustering of scores in the high end of the scale (typically 5-7, or 6-7), without “disagreement” (i.e., scores in the low end of the scale, 1-3). Because we had three axes (confidence in correctness, need, feasibility), we decided to keep or reject each recommendation in two steps. Step 1 would be to assess consensus for each axis. Step 2 would be to make a recommendation selection based on all three axes. However, given the large number of starting recommendations, this two-step process was not applied at the initial recommendation stage. Instead, we relied on rough-cut criteria for consensus as determined by average scores across all axes equal to or greater than 5. We choose this criterion because of the large number of recommendations and the purpose of the first round as a throughput screening device.

To provide some flexibility, we designated 2 sets of criteria – one primary and one secondary (as per RAND), both discussed below. The primary criteria are meant to find consensus across all three domains, rather than simply one. We used the following primary criteria for determining “high (positive) consensus” and “low (negative) consensus”:

- “Positive consensus” - After discarding 1 extreme high and one extreme low rating, there must have been at least 10 ratings ≥ 6, and not more than 5 ratings < 5.
- “Negative consensus” is the inverse, i.e., after discarding 1 extreme high and one extreme low rating, among the remaining ratings, there must have been at least 10 ratings < 5, and not more than 5 ratings ≥ 6.

The secondary criteria applied only to Correctness. We used the following Secondary Criteria for determining “high consensus” and “low consensus”:

- “Positive consensus” - After discarding 1 extreme high and one extreme low rating, among the remaining ratings, there must have been at least 10 ratings ≥6, and not more than 2 ratings < 5.
- “Negative consensus” is the inverse, i.e., after discarding 1 extreme high and one extreme low rating, among the remaining ratings, there must have been at least 10 ratings <5, and not more than 2 ratings ≥ 6.

We used the secondary criteria to ensure representative responses. Because the primary criteria provided a narrower band of recommendations by screening out those that do not have consistent results across responses, they may produce a narrow band of recommendations that are not representative of the breadth of issues involved. Because the crux of consensus requires that the recommendation be correct, not that it be needed or feasible, secondary criteria were used to ensure representativeness.

The secondary criteria could be invoked if the investigators wished to include recommendations that are related and are close to consensus on the primary criteria. Assume, for example, with a full 19 valid responses, that 17 participants agreed it was highly correct and needed, but only 13 participants agreed it was feasible. It would not meet the primary criteria. Assume also that there seems to be a high degree of consensus on two of the three axes, and the third axis represents an area of disagreement that relates to the *practicality* of the recommendation, not to whether it should be pursued. We might still value those responses because the participants who scored the recommendation at 6 were ambivalent or uncertain in their opinions during the survey or meeting or were made up of people who consistently gave lower scores—and because people whose opinions we valued highly said it was highly correct and needed.

### Defining consensus, using selection criteria, and endorsement

We used the primary and secondary criteria to determine consensus in each round. For each recommendation, we used 3 axes for each question in round 1 (importance, correctness, clarity) and round 2 (correctness, need, feasibility). We used the axis of correctness as the main axis to base an endorsement of recommendations.

Because each of the rounds had different subcategories and were directed to different tasks, we modified consensus criteria for each round. In the first, or initial, round of the Delphi, we proposed 119 total recommendations. Since the goal of the Delphi was to develop a smaller set of recommendations, the first round focused on winnowing recommendations down to a more manageable number. Our initial criteria for determining whether a recommendation reached consensus was based on the exclusion of low-scoring recommendations ≤ 3 and including high-scoring recommendations ≥ 5.

“Positive consensus” in Round 1 meant the following: After discarding 1 extreme high and one extreme low rating, among the remaining ratings, there must have been at least 10 ratings ≥ 5, and not more than 2 ratings in < 3. “Negative consensus” in the first round meant the inverse: i.e., after discarding 1 extreme high and one extreme low rating, among the remaining ratings, there must have been at least 10 ratings < 3, and not more than 2 ratings in the between 5 and 7.

In round 1, we used primary criteria applied to Correctness, Need, and Feasibility. A recommendation was selected if there as a high (positive) consensus on all three axis as measured by an aggregate score of 5 or higher.

Round 2 of the Delphi contained a smaller number of recommendations. And because these were recommendations on which there was already a high level of agreement, we expected consensus criteria to be narrow. We modified our definition of consensus for round 2 because the responses generated by Round 1 indicated scores of greater than 5, on average. For this reason, we generated higher cutoff criteria for what counted as positive and negative consensus.

In round 2, we used a two-part decision framework. A recommendation was selected if there was high (positive) consensus on correctness, need, and feasibility (*using primary criteria*). A recommendation might be selected if there was high (positive) consensus on correctness, (*using secondary criteria*) as long as there is no negative consensus on either need or feasibility (*using strict criteria*).

## Results

### Overview

Twenty-nine recommendations met the prespecified criteria for consensus in the Delphi Process. We summarize them here, organized by the five domains identified earlier.

### Domain 1: recommendations addressing guidelines, certification, and training relating to the use of digital diagnostics

The Delphi Panel achieved consensus on six recommendations addressing the introduction and implementation of guidelines, certifications, and training for use of digital diagnostics. For this domain, the key actors identified were manufacturers, with a downstream focus on patients and practitioners. These recommendations include the need for physicians to understand various demographic factors of their patient population to observe how these influence the patient’s use of the digital diagnostic; the need for manufacturers to develop training tools for physician and non-physician practitioners; and the need for manufacturers to provide patients with easy to understand instructions and on-demand resources which could direct them to videos or visual diagrams on the use of digital diagnostics and in turn, give patients access to comprehensive informational materials.

The Delphi Working Group adopted the following recommendations in this domain:

Recommendation 1: Physicians should use reasonable efforts to understand how patient population (e.g., age, race, socioeconomics) may influence the use of the digital diagnostic.

Recommendation 2: Manufacturers marketing digital diagnostics should develop training tools that should be available to non-physician practitioners who use digital diagnostics.

Recommendation 3: Manufacturers marketing digital diagnostics should develop training tools that should be available to physicians who use digital diagnostics.

Recommendation 4: Manufacturers should provide to patients easy-to-understand instructions for the use of the manufacturers’ digital diagnostics.

Recommendation 5: Manufacturers should provide to patients access to on-demand resources (e.g., a QR code that points to videos, visual depictions/diagrams) for the proper use of the manufacturers’ digital diagnostics (e.g., instructions on use, limitations, etc.).

Recommendation 6: Patients should have access to comprehensive informational materials about the digital diagnostic they use.

### Domain 2: recommendations addressing liability arising from the use of digital diagnostics

The panel endorsed five recommendations addressing the liability arising from the use of digital diagnostics. For this domain, the key actors identified were healthcare organizations and healthcare practitioners, with a downstream focus on product users and caregivers. The recommendations include the need for healthcare organizations such as hospitals and medical centers to develop policies and procedures to monitor and remedy adverse events that arise from use of digital diagnostics; the need for healthcare practitioners to inform patients who use digital diagnostics of any privacy concerns arising out of that use, the patient’s responsibilities while using digital diagnostics, and any risks involved with such use; and the need for healthcare practitioners to also inform caregivers of any risks involved with such use of digital diagnostics.

The Delphi Working Group adopted the following recommendations in this domain:

Recommendation 7: Healthcare organizations, such as hospitals and academic medical centers, that use digital diagnostics should develop a template of policies and procedures for monitoring adverse events associated with the use of digital diagnostics (assuming information from digital diagnostics flows directly to them instead of manufacturers).

Recommendation 8: Healthcare practitioners that use digital diagnostics should inform patients about the privacy concerns arising from the use of digital diagnostics that the manufacturer communicates to the healthcare practitioner.

Recommendation 9: Healthcare practitioners that use digital diagnostics should adequately inform patients about the patients’ responsibilities when using digital diagnostics.

Recommendation 10: Healthcare practitioners that use digital diagnostics should adequately inform patients about any risks involved in using digital diagnostics.

Recommendation 11: Healthcare practitioners that use digital diagnostics should adequately inform caregivers about any risks involved in using digital diagnostics.

### Domain 3: recommendations addressing the regulation and marketing of digital diagnostics

The panel achieved consensus on six recommendations addressing the regulation and marketing of digital diagnostics. For this domain, the key actors identified were FDA and product manufacturers, with a downstream focus on product users. These recommendations address the reality that some digital diagnostics are regulated by FDA and others are not, a state of affairs the panel thought should persist. The recommendations in this domain include the need for manufacturers to use representative demographic characteristics based on data of the target population to validate products that are regulated by FDA; the need for manufacturers to develop a consumer-friendly template that explains the uses of the digital diagnostics for products not regulated by FDA; the need for FDA to regulate all digital diagnostics it reviews for analytical and clinical validity; the manufacturers’ role in not implying or suggesting any uses other than those that the FDA approves, clears, or authorizes for digital diagnostics regulated by FDA; and the need for manufacturers to develop a uniform consumer-friendly disclosure that explains the uses and limitations of the digital diagnostics for those regulated by the FDA.

The Delphi Working Group adopted the following recommendations in this domain:

Recommendation 12: For digital diagnostics regulated by FDA, manufacturers should use representative data—by including individuals with representative demographic characteristics (e.g., sex, race, age, socioeconomics) of the digital diagnostic’s target population—to validate their products before and after any FDA approval, clearance, or authorization.

Recommendation 13: For digital diagnostics not regulated by FDA, manufacturers should develop a consumer-friendly template that explains the uses of the product.

Recommendation 14: FDA should attempt to evaluate all digital diagnostics it reviews for analytical and clinical validity.

Recommendation 15: For digital diagnostics regulated by FDA, manufacturer marketing should not imply or suggest uses other than those that FDA has approved, cleared, or authorized.

Recommendation 16: For digital diagnostics regulated by FDA, manufacturers should develop a uniform consumer-friendly disclosure that explains the uses of the product.

Recommendation 17: For digital diagnostics regulated by FDA, manufacturers should develop a uniform consumer-friendly disclosure that explains the limitations of the product.

### Domain 4: recommendations addressing reimbursement of digital diagnostics

Effective digital diagnostics will not reach many of the patients that need them absent reimbursement by public and private payers. For this domain, the key actors identified were insurance providers, with a downstream focus on members or prospective members. While some patients might pay for them out of pocket, unless attention is focused on payment, we may see use of these products stymied by a lack of access to digital health technologies, especially in low-income communities and those without insurance coverage. Under this domain, the Delphi panel endorsed three recommendations addressing reimbursement of digital diagnostics. These recommendations include the need for Centers for Medicare and Medicaid Services (CMS) to articulate specific criteria for reimbursement that proactively assess digital diagnostics; the need for private insurance companies to explain to their members in plain language their expected financial responsibility under the member’s policy for payment due for digital diagnostics; and the obligation on private insurance companies to develop clear policies for a reimbursement procedure for digital diagnostics.

The Delphi Working Group adopted the following recommendations in this domain:

Recommendation 18: The Centers for Medicare and Medicaid Services (CMS) should articulate specific criteria for reimbursement that proactively assess digital diagnostics (beyond parallel review).

Recommendation 19: Private insurance companies should explain to their members in plain language the member’s expected financial responsibility for a digital diagnostic paid for under the member’s policy.

Recommendation 20: Private insurance companies should articulate and develop clear policies on their reimbursement procedure for digital diagnostics.

### Domain 5: recommendations addressing privacy, security, and consent in the use of digital diagnostics

Digital Diagnostics implicate privacy, security, and consent because they will collect information on patients, raising the possibility that the information could be hacked or shared by manufacturer without patient knowledge or consent. For this domain, the key actors identified were manufacturers, stakeholders, patients, and the Federal Trade Commission (FTC), with a downstream focus on product users. The panel achieved consensus on nine recommendations addressing privacy, security, and consent in the use of digital diagnostics. These include the need for manufacturers to develop consensus on technical standards for digital diagnostics; the advice that manufacturers should use privacy-by-design principles—building in specific privacy controls, such as defaults, into the design of the product, rather than trying to regulate them solely *after* development; the need for stakeholders to convene to develop model ethical principles that prioritize the privacy and security of patient data; the right of patients to have access to data collected by digital diagnostics; the need for manufacturers to develop manuals that help patients understand in plain language how their information is stored, collected, and used; the advice for manufacturers to provide consumer-friendly disclosures detailing how consumers can protect their information; and the need for FTC to play a more active role in regulating false and misleading advertising claims for digital diagnostics and maintaining consumer privacy by regulating data breaches.

The Delphi Working Group adopted the following recommendations in this domain:

Recommendation 21: To ensure accessible deployment of technology across patient populations and settings, manufacturers should adopt consensus on technical standards for digital diagnostics.

Recommendation 22: Manufacturers should use privacy-by-design principles to develop products with privacy protections “built in” to the product’s functionality.

Recommendation 23: Stakeholders (e.g., manufacturers, ethicists, and physicians) should convene to develop model ethical principles for the design, implementation, and use of digital diagnostics that prioritize the privacy of patient data.

Recommendation 24: Stakeholders (e.g., manufacturers, ethicists, and physicians) should convene to develop model ethical principles for the design, implementation, and use of digital diagnostics that prioritize the security of patient data.

Recommendation 25: Patients should have a meaningful right to access information collected by a digital diagnostic

Recommendation 26: Manufacturers should develop easy-to-understand instruction manuals that help patients understand in plain language how their information is gathered, stored, and used.

Recommendation 27: Manufacturers should provide consumer-friendly disclosures about how they protect consumer information.

Recommendation 28: The Federal Trade Commission (FTC) should maintain an active presence in the digital health space to police false and misleading advertising claims.

Recommendation 29: The Federal Trade Commission (FTC) should continue to protect consumer privacy using its authority to regulate data breaches.

## Discussion

Like any other emerging technologies, digital diagnostics generate a number of legal and ethical issues with no immediate or simple answer. One such area is liability, which may arise from the development, design, prescription, or use of digital diagnostics. The recommendations respond to this concern by ensuring all relevant parties that interact with digital diagnostics are reasonably informed about the diagnostic’s capabilities and operation (1-5). This is important because liability risks that stem from using these devices extend beyond physicians to patient and caregivers as well^18^. For example, a caregiver may be injured at a patient’s home while assisting with a digital diagnostic. Or they may incorrectly assist the patient using the digital diagnostic, causing a false positive or negative that results in injury.

The participants recognized that the legal and ethical responsibilities should be commensurate with available information, including by placing a greater burden on manufacturers to provide information than on physicians or patients to ferret it out on their own (which in many cases is actually or practically impossible) (7-9). The participants did not reach consensus on two recommendations that would have required patients and caregivers to take a short quiz on how to use the digital diagnostic prior to using them. Participants also did not reach consensus on a recommendation that would have required digital diagnostic trainings to be a required element of clinician licensure training or education. Conversely, participants also did not endorse a recommendation to limit liability entirely, failing to reach consensus on amending laws to provide additional protection for physicians that rely on digital diagnostics.

Participants did not endorse recommendations that would have directly shifted liability to healthcare organizations, which may engage in hospital-at-home programs that require patients and caregivers to use digital diagnostics (8-11). They did, however, recognize that these institutions have a significant role to play (7). Participants reached consensus on a recommendation for such providers to develop template policies to be used at a variety of institutions, perhaps even formalizing a custom that liability law would take into account when making liability determinations^19^. While the Delphi participants did not discuss the details about how exactly to effectuate such a move, hospitals and large academic medical centers, accrediting organizations, like the Joint Commission, might formulate template policies that could be customized to meet the needs of individual institutions, and tort law might look at these templates for guidance.

Open questions also remain to how federal and state regulators should confront digital diagnostics. While Delphi participants did not reject FDA’s current approach to regulation, they recognized that more was needed in some key areas. The recommendations broadly supported FDA’s continued enforcement of regulatory controls on digital diagnostics it reviews, along with clinical and analytical validation that uses representative (demographic) data. Moreover, several recommendations also embraced FDA’s current approach, driven by its statutory authority, that exempts certain types of products categories from FDA regulation (12-15). What separates these “general wellness products” from devices seems clear on paper but can often be a difficult line for FDA to police^19^. The recommendations generally supported some flexibility in FDA’s approach, and also suggested manufactures develop a uniform consumer-friendly disclosure to help inform those using the product (13, 16-17). There are existing proposals relating to off-label uses of drugs^20^, nutrition labels for AI^21^, and health apps^22^ that could be adapted by FDA or private industry to effectuate these recommendations.

FDA is not the only agency active in this space. FTC’s regulation of advertising is also important. The participants endorsed recommendations focused on FDA rather than FTC, but this may be an artifact of it being the agency with which they were more familiar. Indeed, in the first-round participants endorsed recommendations regarding continuing FTC enforcement and potential enforcement expansion with high consensus. But the strict criteria for consensus in the second round meant these recommendations did not advance to subsequent rounds. FTC was also discussed more in the context of consumer privacy (29), probably owing to FTC’s ongoing presence and public attention in the privacy space.

For digital diagnostics to meaningfully improve the health of all patients, it is essential that there be some form of reimbursement for their use. The participants recommended that insurers have clear policies about covering and reimbursing digital diagnostics (18-20). Consumer-friendly tools to help insurance members understand and estimate costs were also recommended both before and after insurance selection by a customer. Indeed, insurers already have models they can adapt to this purpose. For example, Medicare has an online tool that helps customers estimate plan and drug costs, including customizing for the drugs a consumer already takes^23^. In the private insurance space, insurers already offer tools to determine whether physicians are in network. A recent federal law has required providers to give estimates before service is rendered, though its effect has thus far not been very pronounced^24^. Similar efforts could be made by public and private insurers with respect to coverage and cost of digital diagnostics. Participants did not reach the question of how pricing tools may be linked to other information about the patient, including information derived from digital diagnostics and other sources like consumer electronics (like a smart refrigerator or Amazon Alexa)^25^, an issue worthy of further discussion.

Beyond the use of data *for* pricing, the participants also considered collection, disclosure, sale, and use of data collected by digital diagnostics. Their recommendations here emphasized the importance of privacy, security, and consent. The general thrust of the recommendations the participants endorsed is that manufacturers and developers ought to build in guardrails by incorporating ethical principles in the design of their products, with particular emphasis on privacy and security (22-24). Others have suggested similar approaches to incorporating ethical principles into the design of the product rather than trying to police privacy and security through amorphous notions of consent after the product is already on the market^26^. Wariness about reliance on consent was also reflected in the fact that the participants failed to reach consensus on a recommendation that would require physicians to seek “re-consent” each time a manufacturer of a digital diagnostic updated its software in a way that significantly affects the functioning of the product. In the first round the participants supported a recommendation (25) for a patient’s universal and meaningful right to access their data with relatively strong consensus, but the strict criteria applied in Round 2 meant that this recommendation, though still strongly supported, did not meet the criteria to advance to Round 3.

The participants also identified the importance of the individual patient as decision-making agent. The relevant recommendations emphasized different aspects of agency, ranging from information about what happens with their data (26-27) to a right to access the data collected by the digital diagnostic (25) to ensuring technical standards enable access to choose in the first instance (21). FTC enforcement, too, was seen to be an important component of this agency, ensuring accurate information reaches consumers and companies are held accountable for data breaches (28-29).

This study has several limitations. First, like any Delphi, the type and content of recommendations are neither exhaustive nor random. The study authors developed potential recommendations based on research and consultation with others working in the area, but this did not capture all potential recommendations or all possible domains or necessarily a representative sample of them. Second, the high level of agreement on almost all potential recommendations suggests that the study did not capture recommendations that may be strongly disfavored. For example, the study did not recommend privatizing part or all of regulatory framework for digital diagnostics. This recommendation may have received strong negative consensus, but it was not evaluated. Third, the selection criteria used to accept recommendations required extremely high levels of agreement, suggesting that while some of the recommendations not selected also may be helpful to policymakers, the ones selected deserve priority in consideration. Fourth, while we were able to recruit participants representing a very diverse set of stakeholders, there were some interests in this area we did not succeed in recruiting for the Delphi. For instance, our study did not include a technology entrepreneur who might engage in serial development of digital diagnostics. Our study also did not include any participant from a large device manufacturer or large institutional investor. This may have resulted in high consensus for some recommendations that would have achieved lower, no, or negative consensus had additional individuals been included. Fifth, many of the recommendations that failed to reach consensus did so because participants rated them low on feasibility. This suggests that the results may be biased in favor of recommendations that, while feasible, may not be reflective of the breadth of concerns participants thought was important to consider.

As investment in and rollout of in-home digital diagnostic gains steam, developers, regulators, and public and private insurers are all trying to formulate policy in this space. Our Delphi process brought together 19 experts with diverse experience—including founders, academics, practicing lawyers at leading technology and insurance companies, physicians, and entrepreneurs— who endorsed 29 consensus recommendations we believe should help set the agenda in five domains: (1) guidelines, certification, and training relating to the use of digital diagnostics; (2) liability arising from the use of digital diagnostics; (3) the regulation and marketing of digital diagnostics; (4) reimbursement of digital diagnostics; and (5) privacy, security, and consent in the use of digital diagnostics.

Our results indicate the need of increased involvement across a diverse portfolio of stakeholders such as physicians, legislators, regulators, manufacturers, public and private insurance companies, healthcare practitioners and providers, and patients and caregivers to better understand and integrate digital diagnostics in the healthcare system. Because of the diverse range of stakeholders, the recommendations varied according to what actions each stakeholder should take.

Nevertheless, there are several key takeaways from the study. First, the results an emphasize a need to provide simple, understandable information about digital diagnostics to patients and physicians. This should include additional resources beyond product labeling that instruct individuals on how to use the device, as well as its limitations and risks. Second, physicians and policy makers should develop guidelines and standards for using digital diagnostics that provide a framework for assessing liability when the digital diagnostic or the physician using one cause harm to a patient. Third, regulators and manufacturers have a key role to play, not only in providing information, but also in ensuring that the digital diagnostic works safely and effectively for the target population and condition. Fourth, reimbursement should be a key element of digital diagnostics policy, both as a tool to ensure equitable access and to incentivize innovation of new technologies. Fifth, digital diagnostic policy should include rules of data management and security that protect patient information but also allow for the interoperability of digital diagnostics across platforms and the sharing of data for research and innovation purposes.

These recommendations reflect a diverse range of issues that digital diagnostics implicate. And while policymakers should consider these as important components of any strategy, they should not be viewed as conclusive of all concerns appliable to digital diagnostics. Nor should they be viewed as exhaustive of the potential recommendations for all digital diagnostics. Despite their limitations, however, these recommendations provide a framework for policymakers thinking about concerns, challenges, and opportunities that digital diagnostics raises. And they should be carefully considered as technology advances.

### Supplementary information

Supplemental Information

## Data Availability

The datasets used and/or analyzed during the current study available from the corresponding author on reasonable request.
