# Supplementary material for: Using digital technologies to diagnose in the home: recommendations from a Delphi panel
Source: NPJ Digit Med. 2024 Jan 22;7:18. doi: 10.1038/s41746-024-01009-8 (PMC10803339; doi:10.1038/s41746-024-01009-8)
Supplement: Supplementary file 1 — Supplemental Information [file 41746_2024_1009_MOESM1_ESM.docx]

Supplementary Table 1. Delphi Working Group Participants and Affiliations

| Name | Affiliation |
| --- | --- |
| Aakash Shah | CEO, Wyndly |
| Aaron Goldmuntz | Chief Operating Officer, Center for Medical Interoperability |
| Bernard Lo | Professor Emeritus, UCSF |
| Brian Williamson | Former Health Products Counsel, Formerly Google |
| Charlie Crawford | Vice President, Chrysalis |
| Charlotte Tschider | Assistant Professor, Loyola University Chicago School of Law |
| Diane Reidy | Vice Chair DOM, MSKCC |
| Giorgio Quer | Director of AI, Scripps Research |
| Jonelle Saunders | Adjunct Professor, American University Washington College of Law |
| Kayte Spector-Bagdady | Assistant Professor, University of Michigan |
| Megann Vaughn Watters | VP, New Ventures & Strategic Alliances, Laboratory Corporation of America Holdings |
| Michael Caljouw | Vice President, BCBSMA |
| Michael D Abramoff | Professor, University of Iowa |
| Nathan Cortez | Professor, SMU |
| Prashant Vaishnava | Senior Medical Director, Cardiovascular Care, Biofourmis |
| Rowan Chapman | Founder/CEO, Initiate Studios |
| Tanya Zvonkin | PFAQC, MSK |
| Timothy Sielaff | Senior Medical Advisor, Inbound Health |
| Vijay Patel | Managing Partner, CVS Health Ventures |

Supplementary Table 2. Recommendations Proposed to Participants in Round 1

| Guidelines, Certification, and Training | |
| --- | --- |
| Medical Organizations | 1. Professional medical organizations, such as the American Academy of Family Medicine, should play an active role in developing guidance for physicians who encounter digital diagnostics in their respective practice areas (such as oncology, pediatrics, pain management, etc.). |
|  | 1. Medical societies and related organizations (such as the Digital Medicine Society (DiMe), American Telemedicine Association, American Medical Informatics Association, American Nursing Informatics Association, and/or the Certification Commission for Health Information Technology etc.) should develop certification programs for digital diagnostics that certify individuals who have received training in use of such technologies, which meets standards set by the organization. |
|  | 1. Assuming certification programs for digital diagnostics as discussed above are developed, they should be available to physicians. |
|  | 1. Assuming certification programs for digital diagnostics as discussed above are developed, they should be available to non-physician practitioners. |
|  | 1. Board certification organizations, such as the American Board of Internal Medicine, should implement digital diagnostics literacy as part of their certification requirements. |
|  | 1. Licensing boards should require training in digital diagnostics as part of licensure requirements for physicians. |
|  | 1. Hospitals and large medical centers should develop training modules, policies, and procedures that govern the use of digital diagnostics. |
| Physicians | 1. Assuming physicians should obtain certifications for digital diagnostics, physicians should be required to renew certification periodically. |
|  | 1. Physicians who obtain certain certifications for the use of digital diagnostics should receive some immunity from liability for harms caused by the use of digital diagnostics. |
|  | 1. Physicians should be trained in the use of digital diagnostics. |
|  | 1. Physicians should understand the risks and benefits of a digital diagnostic before using it. |
|  | 1. Physicians should understand how the risks and benefits of a digital diagnostic may change over time. |
| Manufacturers | 1. Manufacturers marketing digital diagnostics should develop training tools that should be available to physicians who use digital diagnostics. |
|  | 1. Manufacturers marketing digital diagnostics should develop training tools that should be available to non-physician practitioners who use digital diagnostics. |
|  | 1. Manufacturers should provide to patients easy to understand instructions for the use of the manufacturers’ digital diagnostics. |
|  | 1. Manufacturers should provide to patients access to on-demand resources (e.g., a QR code that points to videos, visual depictions/diagrams) for the proper use of the manufacturers’ digital diagnostics (e.g., instructions on use, limitations, etc.). |
| Patients | 1. Prior to using a digital diagnostic, patients should be required to complete a short training video/instruction course that guides them on how to use the relevant digital diagnostic. 2. Prior to using a digital diagnostic, patients should read all labeling associated with the relevant digital diagnostic. |
|  | 1. Prior to using a digital diagnostic, patients should be required to “pass” a short quiz on how to use the relevant digital diagnostic. |
|  | 1. Patients should have access to comprehensive informational materials about the digital diagnostic they use. |
| Caregivers | 1. Paid or unpaid caregivers should be required to undergo training before using digital diagnostics to care for a patient. |
|  | 1. Paid or unpaid caregivers should be ethically required to understand their role in assisting the patient with a digital diagnostic (e.g., fitting the product, monitoring its placement or battery life). |
|  | 1. Paid or unpaid caregivers should be legally required to understand their role in assisting the patient with a digital diagnostic (e.g., fitting the product, monitoring its placement or battery life). |
|  | 1. Prior to using a digital diagnostic, paid or unpaid caregivers should be required to “pass” a short quiz on how to use the relevant digital diagnostic. |
| Licensing/Accreditation & Standard Setting Bodies | 1. State licensure bodies should consider adding a requirement on digital literacy for physicians using digital diagnostics (e.g., Continuing Medical Education (CME)). |
|  | 1. State licensure bodies should consider adding a requirement on digital literacy for hospitals using digital diagnostics (e.g., training courses). |
|  | 1. Accrediting bodies, like the Liaison Committee on Medical Education, should incorporate digital diagnostic education and training requirements into the accreditation process. |
|  | 1. Standard-setting organizations should work to develop universal interoperability standards for digital diagnostics to enable them to work together. |
|  | 1. Standard-setting organizations should work to develop universal interoperability standards for digital diagnostics to enable them to work together without the need for private agreements (e.g., licensing). |
|  | 1. Standard-setting organizations should identify interoperability standards to be used across all digital diagnostics platforms. |
| Liability | |
| Legislators | 1. Legislators should revise liability standards to protect physicians from a malpractice lawsuit arising from the use of a digital diagnostic unless the patient suing the physician can prove that the digital diagnostic is reliable. |
|  | 1. Legislators should revise liability standards to protect physicians from a malpractice lawsuit arising from the use of a digital diagnostic unless the patient suing the physician can prove that the digital diagnostic is analytically valid. |
|  | 1. Legislators should revise liability standards to protect physicians from a malpractice lawsuit arising from the use of a digital diagnostic unless the patient suing the physician can prove that the digital diagnostic is clinically valid. |
|  | 1. If FDA does not review a digital diagnostic for safety and effectiveness, malpractice liability for physicians who use information from digital diagnostics should be treated the same as patient-reported data (i.e., information that is reported directly by patients to their physician). |
|  | 1. If FDA does review a digital diagnostic for safety and effectiveness, malpractice liability for physicians who use information from digital diagnostics should be treated the same as patient-reported data (i.e., information that is reported directly by patients to their physician). |
| Healthcare Practitioners & Providers | 1. Healthcare organizations, such as hospitals and academic medical centers, that use digital diagnostics should have clear policies and procedures in place for monitoring adverse events associated with the use of digital diagnostics (assuming information from digital diagnostics flows directly to them instead of manufacturers). |
|  | 1. Healthcare organizations, such as hospitals and academic medical centers, that use digital diagnostics should have clear policies and procedures in place for undertaking corrective actions based on adverse events reported from the use of digital diagnostics. |
|  | 1. Healthcare practitioners who use digital diagnostics by contracting with or working through third parties should clearly inform patients about these contractual relationships. |
|  | 1. Healthcare practitioners who use digital diagnostics by contracting with or working through third parties should clearly inform patients about the responsibilities of each party under the terms of the agreement. |
|  | 1. Healthcare practitioners that use digital diagnostics should adequately inform patients about the limits of the technology. |
|  | 1. Healthcare practitioners that use digital diagnostics should adequately inform patients about any risks involved in using digital diagnostics. |
|  | 1. Healthcare practitioners that use digital diagnostics should adequately inform patients about privacy concerns arising from the use of digital diagnostics. |
|  | 1. Healthcare practitioners that use digital diagnostics should adequately inform patients about the patients' responsibilities when using digital diagnostics |
|  | 1. Healthcare practitioners that use digital diagnostics should adequately inform caregivers about the limits of the technology. |
|  | 1. Healthcare practitioners that use digital diagnostics should adequately inform caregivers about any risks involved in using digital diagnostics. |
|  | 1. Healthcare practitioners that use digital diagnostics should adequately inform caregivers about privacy concerns arising from the use of digital diagnostics. |
|  | 1. Healthcare practitioners that use digital diagnostics should adequately inform caregivers about the caregivers' responsibilities when using digital diagnostics. |
|  | 1. Healthcare practitioners that use digital diagnostics should adequately inform caregivers about the responsibilities of patients who use digital diagnostics. |
| Caregivers | 1. Caregivers should communicate regularly with the care team about whether the digital diagnostic is functioning properly (i.e., whether it is working, whether it is turned on properly). |
|  | 1. Caregivers should orally communicate to the patient the caregiver’s role in the patient’s care. |
|  | 1. Caregivers should in writing communicate to the patient the caregiver’s role in the patient’s care. |
| Regulation and Marketing | |
| Regulation and Marketing | 1. Regulators should continue to take a relaxed regulatory approach (i.e., exercise enforcement discretion over) towards technologies that are low-risk general wellness products. |
|  | 1. Regulators should continue to experiment with new models of regulation—such as [FDA’s Pilot PreCert](https://www.fda.gov/medical-devices/digital-health-center-excellence/digital-health-software-precertification-pre-cert-pilot-program) program, which sought to regulate the device lifecycle, rather than the marketed device—for moderate- to high-risk digital diagnostics. |
|  | 1. Regulators should work to develop additional consumer-friendly labeling that provides basic information about a digital diagnostic (e.g., instructions on use, training data used to validate, limitations on use). |
|  | 1. Regulators should reduce regulatory burdens for small- and medium-sized manufacturers of digital diagnostics seeking to bring novel devices to market. |
|  | 1. Regulators should experiment with programs that enable innovative manufacturers to move from unregulated to regulated devices with varying degrees of FDA oversight. |
|  | 1. Congress should give FDA authority to regulate digital diagnostics for analytical validity. |
|  | 1. Congress should give FDA authority to regulate digital diagnostics for clinical validity (see, e.g., [VALID Act](https://www.congress.gov/bill/117th-congress/house-bill/4128/text?q=%7B%22search%22%3A%5B%22valid+act%22%5D%7D&r=1&s=1)). |
|  | 1. FDA should evaluate digital diagnostics for analytical validity. |
|  | 1. FDA should evaluate digital diagnostics for clinical validity. |
| Manufacturers | 1. For digital diagnostics not regulated by FDA, manufacturers should use representative data—by including individuals with representative demographic characteristics (e.g., sex, race, age, socioeconomics) of the digital diagnostic’s target population—to validate their products. |
|  | 1. For digital diagnostics regulated by FDA, manufacturers should use representative data—by including individuals with representative demographic characteristics (e.g., sex, race, age, socioeconomics) of the digital diagnostic’s target population—to validate their products. |
|  | 1. For digital diagnostics not regulated by FDA, manufacturer marketing should not imply or suggest uses that may cause patients to believe that the product has some diagnostic function that FDA traditionally regulates by approval, clearance, or authorization. |
|  | 1. For digital diagnostics regulated by FDA, manufacturer marketing should not imply or suggest uses other than those that FDA has approved, cleared, or authorized. |
|  | 1. For digital diagnostics not regulated by FDA, manufacturers should develop a uniform consumer-friendly disclosure that explains the uses of the product. |
|  | 1. For digital diagnostics regulated by FDA, manufacturers should develop a uniform consumer-friendly disclosure that explains the uses of the product. |
|  | 1. For digital diagnostics not regulated by FDA, manufacturers should develop a uniform consumer-friendly disclosure that explains the limitations of the product. |
|  | 1. For digital diagnostics regulated by FDA, manufacturers should develop a uniform consumer-friendly disclosure that explains the limitations of the product. |
|  | 1. For digital diagnostics not regulated by FDA, manufacturers should engage in consumer awareness education about the limits of digital diagnostics. |
|  | 1. For digital diagnostics regulated by FDA, manufacturers should engage in consumer awareness education about the limits of digital diagnostics. |
|  | 1. For digital diagnostics regulated by FDA, manufacturers should work with federal regulators to add consumer-friendly labeling to all digital diagnostics regulated by the FDA. |
| Reimbursement | |
| Manufacturers | 1. Manufacturers should collaborate with insurance companies to prospectively provide information to healthcare providers about (estimated or approximate) coverage (as reasonably possible). |
|  | 1. Manufacturers should collaborate with insurance companies to prospectively provide information to consumers about (estimated or approximate) coverage (as reasonably possible). |
|  | 1. Assuming the information in the previous question is provided to consumers, it should be provided to them prior to selecting coverage. |
|  | 1. Assuming the information in the previous question is provided to consumers, it should be provided to them prior to deciding whether to use a digital diagnostic. |
|  | 1. Assuming the information in the previous question is provided to consumers, it should be provided to them prior to purchasing a specific digital diagnostic. |
| Private Insurance | 1. Private insurance companies should articulate and develop clear policies on their reimbursement procedure for digital diagnostics. |
|  | 1. Private insurance companies should explain in plain language to beneficiaries the manufacturer’s reimbursement policies. |
|  | 1. Private insurance companies should not reimburse for use of a digital diagnostic unless there is robust evidence supporting the accuracy of the digital diagnostic. |
|  | 1. Private insurance companies should not reimburse for use of a digital diagnostic unless there is robust evidence supporting the reliability of the digital diagnostic. |
|  | 1. Private insurance companies should not reimburse for use of a digital diagnostic unless there is robust evidence supporting the analytical validity of the digital diagnostic. |
|  | 1. Private insurance companies should not reimburse for use of a digital diagnostic unless there is robust evidence supporting the clinical validity of the digital diagnostic. |
|  | 1. Private insurance companies should develop a “unified insurance database for digital diagnostics” (or novel technologies) or some uniform tool that enables anyone to determine insurance coverage for the product (e.g., similar to what many insurance companies offer with drug pricing tools but for *all* insured). |
| Public Insurance | 1. The Centers for Medicare and Medicaid Services (CMS) should articulate specific criteria for reimbursement that proactively assess digital diagnostics (beyond parallel review). |
|  | 1. The Centers for Medicare and Medicaid Services (CMS) should develop a more streamlined process for obtaining new technology codes (i.e., codes needed to submit claims to CMS for payment). |
|  | 1. The Centers for Medicare and Medicaid Services (CMS) should develop a more streamlined process for reimbursing digital diagnostics with new technology codes. |
|  | 1. The Centers for Medicare and Medicaid Services (CMS) should develop rules that distribute digital diagnostics equitably. For example, digital diagnostics should not be deployed to resource-poor or resource-rich settings simply because it increases provider profit or reduces overall cost. |
|  | 1. The Centers for Medicare and Medicaid Services (CMS) should collect information about the prescribed use of digital diagnostics to its beneficiaries. |
|  | 1. The Centers for Medicare and Medicaid Services (CMS) should collect information about the actual use of digital diagnostics by its beneficiaries. |
|  | 1. The Centers for Medicare and Medicaid Services (CMS) should collect information about the outcomes of its beneficiaries who use digital diagnostics. |
|  | 1. The Centers for Medicare and Medicaid Services (CMS) should initiate a pilot program to determine reimbursement for novel digital diagnostics. |
|  | 1. The Centers for Medicare and Medicaid Services (CMS) should provide to [Medicare Advantage](https://www.medicare.gov/sign-upchange-plans/types-of-medicare-health-plans/medicare-advantage-plans) programs significant flexibility in determining reimbursement strategies for digital diagnostics. |
|  | 1. The Centers for Medicare and Medicaid Services (CMS) should continue to experiment with reimbursement rules that speed access to new technologies. |
|  | 1. The Centers for Medicare and Medicaid Services (CMS) should study whether existing “add-on” payments for new technologies under Medicare are sufficient to drive innovation in digital diagnostics. |
| Privacy / Security / Consent | |
| Stakeholders/Organizations | 1. Stakeholders (e.g., manufacturers, ethicists, and physicians) should convene to develop model ethical principles for the design, implementation, and use of digital diagnostics that prioritize the privacy of patient data. |
|  | 1. Stakeholders (e.g., manufacturers, ethicists, and physicians) should convene to develop model ethical principles for the design, implementation, and use of digital diagnostics that prioritize the security of patient data. |
|  | 1. Stakeholders (e.g., manufacturers, ethicists, and physicians) should convene to develop model ethical principles for the design, implementation, and use of digital diagnostics that prioritize patient consent. |
| Patients | 1. Patients should have a meaningful right to limit the use of the data generated by a digital diagnostic (e.g., rights to be forgotten, data sharing). |
|  | 1. Patients should have a meaningful right to access information collected by a digital diagnostic. |
|  | 1. Patients should have a meaningful right to appropriate their own data for commercial purposes. |
| Manufacturers | 1. Manufacturers should design systems that enable obtaining meaningful consent at multiple points during the use of the product. |
|  | 1. Manufacturers should work to develop technical standards for digital diagnostics that allow for maximum interoperability across health care environments and products. |
|  | 1. Manufacturers should use ethics-by-design principles to develop products with ethical protections "built in" to the product's functionality. |
|  | 1. Manufacturers should use privacy-by-design principles to develop products with privacy protections "built in" to the product's functionality. |
|  | 1. Manufacturers should develop easy-to-understand instruction manuals that help patients understand in plain language how their information is gathered, stored, and used. |
|  | 1. Manufacturers should provide users with the ability to delete or purge data collected by digital diagnostics. |
|  | 1. Manufacturers should provide users with the ability to limit how data collected by a digital diagnostic are used. |
|  | 1. Manufacturers should provide consumer-friendly disclosures about how they protect consumer information. |
|  | 1. Manufacturers should provide consumer-friendly disclosures about how they use consumer information. |
| Physicians | 1. Physicians using and recommending digital diagnostics should develop a model of consent that accounts for the nature and function of the digital diagnostic. |
|  | 1. Physicians using and recommending digital diagnostics should be required to obtain consent after a software change that significantly affects the functioning of the product. |
| Regulators/Legislators | 1. The Federal Trade Commission (FTC) should maintain an active presence in the digital health space to police false and misleading advertising claims. https://www.ftc.gov/news-events/news/press-releases/2021/06/ftc- finalizes-order-flo-health-fertility-tracking-app-shared-sensitive-health-data-facebook-google |
|  | 1. The Federal Trade Commission (FTC) should continue to protect consumer privacy using its authority to regulate data sharing. |
|  | 1. The Federal Trade Commission (FTC) should continue to protect consumer privacy using its authority to regulate data breaches. |
|  | 1. States’ attorney generals should articulate, or continue to articulate, clear and specific policies on the enforcement of deceptive practices and other state laws related to digital diagnostics. |
|  | 1. State regulators should limit third-party access to certain kinds of information generated by digital diagnostics. |
|  | 1. Legislators should create a uniform right of access for consumers whose data is collected digital diagnostic products. |
|  | 1. Legislators should create a uniform right of access for patients whose data is collected digital diagnostic devices. |

Supplementary Table 3. Recommendations Proposed to Participants in Round 2*

*Note: Recommendation numbers in this table correspond to the recommendation number in the table above for Round 1.

| Guidelines and Training | |
| --- | --- |
| Physicians | Recommendation 11: Physicians should understand the risks and benefits of a digital diagnostic before using it. |
|  | Recommendation 12: Physicians should understand how the risks and benefits of a digital diagnostic may change over time. |
|  | Added in Round 2: Physicians should understand how patient population (e.g., age, race, socioeconomics) may influence the efficacy of the digital diagnostic. |
|  | Added in Round 2: Physicians should understand the limitations (e.g., technological, economic) of patient population that may influence the ability of a patient to use the digital diagnostic. |
| Manufacturers | Recommendation 13: Manufacturers marketing digital diagnostics should develop training tools that should be available to physicians who use digital diagnostics. |
|  | Recommendation 14: Manufacturers marketing digital diagnostics should develop training tools that should be available to non-physician practitioners who use digital diagnostics. |
|  | Recommendation 15: Manufacturers should provide to patients easy to understand instructions for the use of the manufacturers’ digital diagnostics. |
|  | Recommendation 16: Manufacturers should provide to patients access to on-demand resources (e.g., a QR code that points to videos, visual depictions/diagrams) for the proper use of the manufacturers' digital diagnostics (e.g. instructions on use, limitations, etc.). |
| Patients | Recommendation 20: Patients should have access to comprehensive informational materials about the digital diagnostic they use. |
| Liability | |
| Healthcare Practitioners & Providers | Recommendation 36: Healthcare organizations, such as hospitals and academic medical centers, that use digital diagnostics should have clear policies and procedures in place for monitoring adverse events associated with the use of digital diagnostics (assuming information from digital diagnostics flows directly to them instead of manufacturers). |
|  | Recommendation 37: Healthcare organizations, such as hospitals and academic medical centers, that use digital diagnostics should have clear policies and procedures in place for undertaking corrective actions based on adverse events reported from the use of digital diagnostics. |
|  | Recommendation 41: Healthcare practitioners that use digital diagnostics should adequately inform patients about any risks involved in using digital diagnostics. |
|  | Recommendation 42: Healthcare practitioners that use digital diagnostics should adequately inform patients about privacy concerns arising from the use of digital diagnostics. |
|  | Recommendation 43: Healthcare practitioners that use digital diagnostics should adequately inform patients about the patients' responsibilities when using digital diagnostics. |
|  | Recommendation 44: Healthcare practitioners that use digital diagnostics should adequately inform caregivers about the limits of the technology. |
|  | Recommendation 45: Healthcare practitioners that use digital diagnostics should adequately inform caregivers about any risks involved in using digital diagnostics. |
| Regulation and Marketing | |
| Regulators/Legislators | Recommendation 54: Regulators should work to develop additional consumer-friendly labeling that provides basic information about a digital diagnostic (e.g., instructions on use, training data used to validate, limitations on use). |
|  | Recommendation 59: FDA should evaluate digital diagnostics for analytical validity. |
|  | Recommendation 60: FDA should evaluate digital diagnostics for clinical validity. |
| Manufacturers | Recommendation 61: For digital diagnostics not regulated by FDA, manufacturers should use representative data—by including individuals with representative demographic characteristics (e.g., sex, race, age, socioeconomics) of the digital diagnostic’s target population—to validate their products. |
|  | Recommendation 62: For digital diagnostics regulated by FDA, manufacturers should use representative data—by including individuals with representative demographic characteristics (e.g., sex, race, age, socioeconomics) of the digital diagnostic’s target population—to validate their products. |
|  | Recommendation 63: For digital diagnostics not regulated by FDA, manufacturer marketing should not imply or suggest uses that may cause patients to believe that the product has some diagnostic function that FDA traditionally regulates by approval, clearance, or authorization. |
|  | Recommendation 64: For digital diagnostics regulated by FDA, manufacturer marketing should not imply or suggest uses other than those that FDA has approved, cleared, or authorized. |
|  | Recommendation 65: For digital diagnostics not regulated by FDA, manufacturers should develop a uniform consumer-friendly disclosure that explains the uses of the product. |
|  | Recommendation 66: For digital diagnostics regulated by FDA, manufacturers should develop a uniform consumer-friendly disclosure that explains the uses of the product. |
|  | Recommendation 67: For digital diagnostics not regulated by FDA, manufacturers should develop a uniform consumer-friendly disclosure that explains the limitations of the product. |
|  | Recommendation 68: For digital diagnostics regulated by FDA, manufacturers should develop a uniform consumer-friendly disclosure that explains the limitations of the product. |
|  | Added in Round 2: For all digital diagnostics, manufacturers should ensure that the digital diagnostics are usable for all relevant patient populations (e.g., patients with blindness, dyslexia, etc.) |
| Reimbursement | |
| Manufacturers | Recommendation 76: Assuming manufacturers collaborate with insurance companies to prospectively provide information to consumers about (estimated or approximate) coverage (as reasonably possible), this information should be provided to consumers prior to purchasing a specific digital diagnostic. |
| Private Insurance | Recommendation 77: Private insurance companies should articulate and develop clear policies on their reimbursement procedure for digital diagnostics. |
|  | Recommendation 78: Private insurance companies should explain in plain language to beneficiaries the manufacturer’s reimbursement policies. |
| Public Insurance | Recommendation 84: The Centers for Medicare and Medicaid Services (CMS) should articulate specific criteria for reimbursement that proactively assess digital diagnostics (beyond parallel review). |
|  | Recommendation 85: The Centers for Medicare and Medicaid Services (CMS) should develop a more streamlined process for obtaining new technology codes (i.e., codes needed to submit claims to CMS for payment). |
|  | Recommendation 86: The Centers for Medicare and Medicaid Services (CMS) should develop a more streamlined process for reimbursing digital diagnostics with new technology codes. |
|  | Recommendation 87: The Centers for Medicare and Medicaid Services (CMS) should develop rules that distribute digital diagnostics equitably. For example, digital diagnostics should not be deployed to resource-poor or resource-rich settings simply because it increases provider profit or reduces overall cost. |
|  | Recommendation 88: The Centers for Medicare and Medicaid Services (CMS) should collect information about the prescribed use of digital diagnostics to its beneficiaries. |
| Privacy/Security/Consent | |
| Stakeholders/Organizations | Recommendation 95: Stakeholders (e.g., manufacturers, ethicists, and physicians) should convene to develop model ethical principles for the design, implementation, and use of digital diagnostics that prioritize the privacy of patient data. |
|  | Recommendation 96: Stakeholders (e.g., manufacturers, ethicists, and physicians) should convene to develop model ethical principles for the design, implementation, and use of digital diagnostics that prioritize the security of patient data. |
|  | Recommendation 97: Stakeholders (e.g., manufacturers, ethicists, and physicians) should convene to develop model ethical principles for the design, implementation, and use of digital diagnostics that prioritize patient consent. |
|  | Added in Round 2: Stakeholders (e.g., manufacturers, researchers, and physicians) should convene to develop uniform guidelines for sharing data from digital diagnostics with researchers. |
|  | Added in Round 2: Stakeholders (e.g., manufacturers, researchers, and physicians) should convene to develop uniform guidelines for generating an accessible database from data generated by digital diagnostics. |
| Patients | Added in Round 2: Patients should have a meaningful right to limit the use of the data generated by a digital diagnostic (e.g., rights to be forgotten, data sharing). |
|  | Added in Round 2: Patients should have a meaningful right to access information collected by a digital diagnostic. |
| Manufacturers | Recommendation 102: Manufacturers should work to develop technical standards for digital diagnostics that allow for maximum interoperability across health care environments and products. |
|  | Recommendation 104: Manufacturers should use privacy-by-design principles to develop products with privacy protections "built in" to the product's functionality. |
|  | Added in Round 2: Manufacturers should develop easy-to-understand instruction manuals that help patients understand in plain language how their information is gathered, stored, and used. |
|  | Recommendation 107: Manufacturers should provide users with the ability to limit how data collected by a digital diagnostic are used. |
|  | Added in Round 2: Manufacturers should provide consumer-friendly disclosures about how they protect consumer information. |
|  | Added in Round 2: Manufacturers should provide consumer-friendly disclosures about how they use consumer information. |
| Regulators/Legislators | Added in Round 2: Congress should enact legislation to protect the privacy of patients using digital diagnostics not covered by the Health Insurance Portability and Accountability Act (HIPAA) |
|  | Recommendation 112: The Federal Trade Commission (FTC) should maintain an active presence in the digital health space to police false and misleading advertising claims.  <https://www.ftc.gov/news-events/news/press-releases/2021/06/ftc-finalizes-order-flo-health-fertility-tracking-app-shared-sensitive-health-data-facebook-google> |
|  | Recommendation 113: The Federal Trade Commission (FTC) should continue to protect consumer privacy using its authority to regulate data sharing. |
|  | Added in Round 2: The Federal Trade Commission (FTC) should continue to protect consumer privacy using its authority to regulate data breaches. |
|  | Added in Round 2: States’ attorney generals should articulate, or continue to articulate, clear and specific policies on the enforcement of deceptive practices and other state laws related to digital diagnostics. |

Supplementary Table 4. Recommendations Proposed to Participants in Round 2 but eliminated from consideration*

*Note: Recommendation numbers in this table correspond to the recommendation numbers in the table above for Round 1.

| Guidelines and Training | |
| --- | --- |
| Physicians | Recommendation 15: Physicians should understand how the risks and benefits of a digital diagnostic may change over time. |
|  | Added in Round 2: Physicians should understand the limitations (e.g., technological, economic) of patient population that may influence the ability of a patient to use the digital diagnostic. |
| Liability | |
| Healthcare Practitioners and Providers | Recommendation 44: Healthcare practitioners that use digital diagnostics should adequately inform caregivers about the limits of the technology. |
| Regulation and Marketing | |
| Regulators/Legislators | Recommendation 54: Regulators should work to develop additional consumer-friendly labeling that provides basic information about a digital diagnostic (e.g., instructions on use, training data used to validate, limitations on use). |
| Manufacturers | Recommendation 61: For digital diagnostics not regulated by FDA, manufacturers should use representative data—by including individuals with representative demographic characteristics (e.g., sex, race, age, socioeconomics) of the digital diagnostic’s target population—to validate their products. |
|  | Recommendation 67: For digital diagnostics not regulated by FDA, manufacturers should develop a uniform consumer-friendly disclosure that explains the limitations of the product. |
|  | Added in Round 2: For all digital diagnostics, manufacturers should ensure that the digital diagnostics are usable for all relevant patient populations (e.g., patients with blindness, dyslexia, etc.) |
| Reimbursement | |
| Manufacturers | Recommendation 76: Assuming manufacturers collaborate with insurance companies to prospectively provide information to consumers about (estimated or approximate) coverage (as reasonably possible), this information should be provided to consumers prior to purchasing a specific digital diagnostic. |
| Public Insurance | Recommendation 85: The Centers for Medicare and Medicaid Services (CMS) should develop a more streamlined process for obtaining new technology codes (i.e., codes needed to submit claims to CMS for payment). |
|  | Recommendation 86: The Centers for Medicare and Medicaid Services (CMS) should develop a more streamlined process for reimbursing digital diagnostics with new technology codes. |
|  | Added in Round 2: The Centers for Medicare and Medicaid Services (CMS) should develop rules that distribute digital diagnostics equitably. For example, digital diagnostics should not be deployed to resource-poor or resource-rich settings simply because it increases provider profit or reduces overall cost. |
|  | Added in Round 2: The Centers for Medicare and Medicaid Services (CMS) should collect information about the prescribed use of digital diagnostics to its beneficiaries. |
| Privacy/Security/Consent | |
| Stakeholders/Organizations | Recommendation 97: Stakeholders (e.g., manufacturers, ethicists, and physicians) should convene to develop model ethical principles for the design, implementation, and use of digital diagnostics that prioritize patient consent. |
|  | Added in Round 2: Stakeholders (e.g., manufacturers, researchers, and physicians) should convene to develop uniform guidelines for sharing data from digital diagnostics with researchers. |
|  | Added in Round 2: Stakeholders (e.g., manufacturers, researchers, and physicians) should convene to develop uniform guidelines for generating an accessible database from data generated by digital diagnostics. |
| Patients | Recommendation 98: Patients should have a meaningful right to limit the use of the data generated by a digital diagnostic (e.g., rights to be forgotten, data sharing). |
| Manufacturers | Recommendation 107: Manufacturers should provide users with the ability to limit how data collected by a digital diagnostic are used. |
| Regulators/Legislators | Added in Round 2: Congress should enact legislation to protect the privacy of patients using digital diagnostics not covered by the Health Insurance Portability and Accountability Act (HIPAA) |
|  | Recommendation 113: The Federal Trade Commission (FTC) should continue to protect consumer privacy using its authority to regulate data sharing. |
|  | Recommendation 115: States’ attorney generals should articulate, or continue to articulate, clear and specific policies on the enforcement of deceptive practices and other state laws related to digital diagnostics. |
